# Detection and identification of small-molecule cracking products formed on pyrolysis of cannabidiol part 1: C_3_ – C_10_ compounds

**DOI:** 10.1186/s42238-026-00442-8

**Published:** 2026-05-01

**Authors:** Niara A. Nichols, A. John Dane, Rabi A. Musah

**Affiliations:** Department of Chemistry, Louisiana State University, Baton Rouge, LA 70803 USA

**Keywords:** CBD, Pyrolysis, Cracking Products, GC × GC – HRMS

## Abstract

**Background:**

Cannabidiol (CBD) is one of over 100 naturally occurring phytocannabinoids in *Cannabis*. While extensive research has evaluated the effects that CBD and other phytocannabinoids have on consumers, less attention has been directed towards studying the health impacts of the compounds formed when cannabinoids are burned. Such an investigation is highly desirable, as smoking remains the most common form of *Cannabis* consumption. Previous research has investigated the structures of CBD pyrolysis products that are of a molecular mass similar to or greater than CBD. However, studies of the lower molecular weight products formed when CBD is pyrolyzed at the temperatures of a burning cigarette are limited. This work investigated the identities of the small-molecule cracking products formed from the pyrolysis of CBD.

**Methods:**

CBD standards were pyrolyzed within the temperature range of 400 °C – 800 °C and analyzed using two-dimensional gas chromatography – high-resolution mass spectrometry. A combination of mass spectral fragmentation patterns, retention index data, GC retention times, and comparison of the profiles of detected compounds with those of authentic standards were used to identify cracking products that eluted during the first 19 min of a 90-min analysis.

**Results:**

This investigation revealed a mixture of pyrolysis products whose complexity increased with increasing temperature. A total of 86 compounds were detected, 81 of which were identified. They spanned the alkane (both cyclic and acyclic), alkene (both cyclic and acyclic), alkyne, substituted benzene, aromatic heterocycle, phenol, and ketone compound classes. Seventy-eight of the identified molecules are reported here for the first time as CBD pyrolysis products. Several are known to have adverse health effects, including genotoxicity and neurotoxicity. Others have been reported to possess health benefits such as anti-inflammatory and antioxidant properties.

**Conclusions:**

The pyrolysis of CBD revealed a complex mixture of 86 C_3_ - C_10_ cracking products across all five temperatures analyzed. The largest number of compounds were formed at 800 °C, while the smallest number of products was observed at 400 °C. The vast majority of these compounds have not been reported in previous studies of CBD pyrolysis. Many of the identified products are known to have impacts on human health.

**Supplementary Information:**

The online version contains supplementary material available at 10.1186/s42238-026-00442-8.

## Background

The compound 2-[(1*R*,6*R*)−6-isopropenyl-3-methylcyclohex-2-en-1-yl]−5-pentylbenzene-1,3-diol, known colloquially as cannabidiol (CBD), is one of over 100 naturally-occurring terpenophenolic compounds biosynthesized by plants in the *Cannabis* genus (Kovalchuk et al. 2020; Sirangelo et al. 2022). These molecules are referred to as phytocannabinoids because they are plant-derived and have a skeletal framework that enables them to bind to endocannabinoid receptors in the central nervous system. The first report on the isolation of CBD in purified form appeared in 1940 (Adams et al. 1940), but its chemical structure was not described until 1963 (Mechoulam and Shvo 1963). This compound has four possible stereoisomers, with the 1*R*,6*R* stereoisomer being the one that is naturally occurring (Rao et al. 2025). It is one of a pair of the most dominant phytocannabinoids in *Cannabis sativa (C. sativa)*, with the second and most famous being its psychoactive constitutional isomer, Δ^9^-tetrahydrocannabinol (Δ^9^-THC) (Flores-Sanchez and Verpoorte 2008), which is classified as a scheduled drug in the U.S. Thus, at the federal level, it is a banned substance that is illegal to sell, buy, distribute, or use. However, at the state level, the legality of Δ^9^-THC varies. At the submission of this report, twenty-four U.S. states had approved marijuana possession for recreational use (with marijuana being classified as *Cannabis spp.* containing greater than 0.3% total THC on a dry weight basis), and forty U.S. states had instituted medical marijuana programs (Lampe et al. 2026). Globally, a number of countries have legalized recreational *Cannabis* use, including Canada, Germany, Uruguay, Thailand, and South Africa (Group CICIW 2024).

While an increasing diversity of food and beverage products are being infused or laced with purified THC and CBD, the primary means of consumer exposure to these compounds is inhalation, because the most common route of ingestion remains the smoking of *Cannabis* cigarettes. There is significant interest in the compounds to which humans are exposed when *C. sativa* is ingested, and this has spurred research into their discovery. Consequently, hundreds of studies focused on the isolation and characterization of compounds contained within the *C. sativa* plant matrix and their potential biological effects have appeared (Rock and Parker 2020; ElSohly et al. 2017; Radwan et al. 2021; Helcman and Šmejkal 2022). However, it has also been recognized that the burning of a *C. sativa* cigarette results in pyrolysis of the molecules contained within the plant material, and that this may result in their conversion into other structures (Moir et al. 2008). In addition to the constitutively present cannabinoids, multiple classes of other compounds have been identified in *C. sativa,* including alkaloids, flavonoids, terpenes, fatty acids, phytosterols, and phenolics among others (Andre et al. 2016; Elhendawy et al. 2019; Huang et al. 2023; Kornpointner et al. 2021; Yadav et al. 2023). It stands to reason that their exposure to heat in the course of being burned would result in a highly complex mixture that contains not only the compounds known to be present in the plant material, but various other products into which they might be converted during pyrolysis. While reports on the chemical characterization of *Cannabis* smoke have appeared (Moir et al. 2008; Fehr and Kalant 1972; Graves et al. 2020; Lee et al. 1976; Tjeerdema 1987; Sparacino et al.1990), the chemical complexity of the plant matrix makes it challenging to determine which constitutively present precursor compounds, upon pyrolysis, are responsible for the formation of new molecules to which the consumer might be exposed. However, to date, attempts to systematically investigate the pyrolysis products of individual *C. sativa*-derived compounds to reveal how they are transformed upon heating, have focused on CBD.

**Fig. 1:**
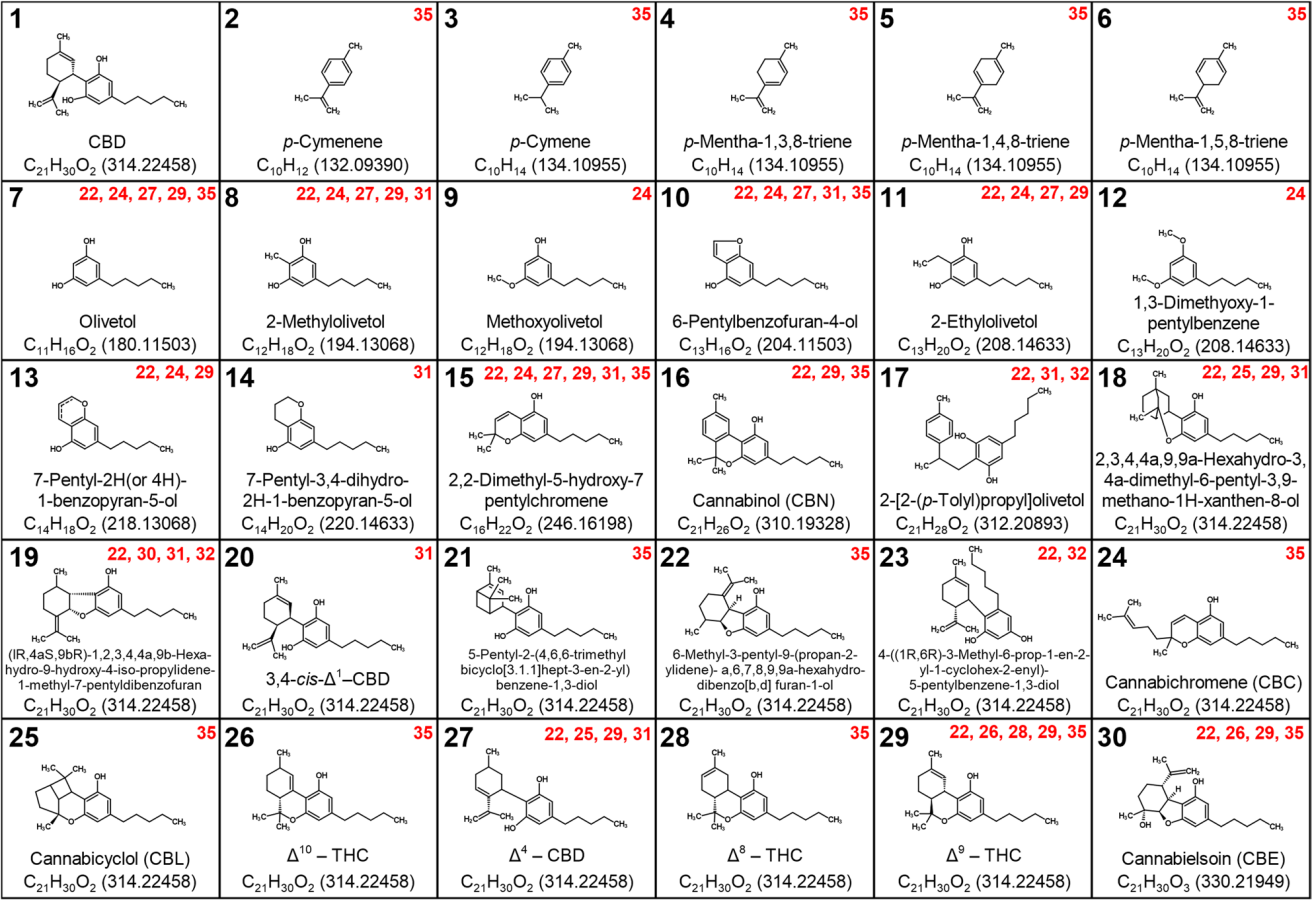
CBD (structure 1) and CBD pyrolysis products (structures 2–30) reported in previous literature, featuring their names, formulas and monoisotopic masses. The literature reference(s) associated with each pyrolysis product is/are shown in the top-right corner in red. The numbers correspond with the references listed in Additional file 3

In a series of reports appearing in the 1970’s and 1980’s (Tjeerdema 1987; Küppers et al. 1975a, 1975b; Küppers et al. 1973; Luteyn et al. 1978; Mikeš and Waser 1971; Salemink and Nahas 1976; Spronck and Lousberg 1977; Spronck et al. 1978; Spronck and Salemink 1978), it was demonstrated that when CBD is pyrolyzed, it is converted into molecules of both higher and lower molecular weight than CBD (Küppers et al. 1975a, 1975b; Küppers et al. 1973). Among the compounds that have been observed are those featured in Fig. 1. One-dimensional gas chromatography (1D GC) and 1D GC-mass spectrometry (MS) were used for the detection and identification of these pyrolysis products, and it was noted that the compounds detected and their relative amounts were dependent upon whether the pyrolysis was conducted in an aerobic or anaerobic environment (Küppers et al. 1975a, 1975b; Salemink and Nahas 1976; Spronck and Lousberg 1977). The results observed under the latter conditions are believed to be more relevant, since research has shown that pyrolysis in burning cigarettes is mainly an anaerobic process. A burning cigarette can reach temperatures as high as 950 °C on the end actively undergoing combustion, and it has been observed that the oxygen content is effectively zero in the gas phase just behind the glowing zone of a burning marijuana cigarette (Tjeerdema 1987; Salemink and Nahas 1976; Bell and Nida 2015; Baker 1975).

Notably, much of the focus in the aforementioned reports was directed towards products with masses similar to or greater than that of CBD, and molecules with a nominal mass range of 132–330 u. However, several of the early investigations highlighted the fact that numerous lower molecular weight compounds that eluted early in the chromatograms were poorly resolved (i.e., they coeluted), and these were never isolated and identified (Küppers et al. 1975a, 1975b; Küppers et al. 1973; Salemink and Nahas 1976). A more recent report (Czégény et al. 2021) has evaluated a fuller range of the compounds produced when CBD undergoes pyrolysis. However, that work was conducted at lower temperatures (250 °C – 500 °C) where the low molecular weight pyrolysis products are less likely to be formed. Thus, studies of the earlier eluting compounds with masses well below that of the starting CBD remain sparse, despite the fact that it would be essential to identify them in order to systematically assess their potential health impacts when ingested.

In this work, we sought to detect and identify the pyrolysis products of CBD with a focus on lower molecular weight compounds eluting early when analyzed by GC, as these were uncharacterized in previous studies due to coelution (Tjeerdema 1987; Küppers et al. 1975a, 1975b; Küppers et al. 1973; Luteyn et al. 1978; Salemink and Nahas 1976; Spronck and Lousberg 1977; Spronck et al. 1978; Spronck and Salemink 1978). We refer to these compounds as “cracking products” because they are formed from the heat-promoted breaking of carbon–carbon bonds in CBD to form lower molecular weight products. Furthermore, we utilized an optimized comprehensive GC × GC – high-resolution mass spectrometry (GC × GC – HRMS) method to detect, resolve, and identify as many of them as possible. Comprehensive GC × GC is a chromatographic technique that uses two different GC columns with different stationary phases connected sequentially to capitalize on their different separation capabilities so that compounds that coelute when using 1D GC are separated from one another in a second dimension through the use of an additional column with different polarity characteristics (Górecki et al. 2004; Mondello et al. 2008). Given that anaerobic conditions are observed immediately following the glow region, this research focused on anaerobic pyrolysis experiments for temperatures up to 800 °C, to model this region of the cigarette where the plant material undergoes anaerobic pyrolysis (Tjeerdema 1987; Salemink and Nahas 1976). Other research on the temperature of burning tobacco cigarettes notes that gas phase constituents reach a maximum temperature at approximately 825 °C just beyond the burning line. Temperatures above 800 °C, which are achieved at the burn line, were not selected for this study, in order to avoid the complete combustion and breakdown of CBD into carbon dioxide and water, which is observed in the combustion region. Additionally, temperatures below 400 °C were not the focus of this work, as thermal desorption, as opposed to bond cleavage, primarily occurs below this temperature (Baker 1975).

## Methods

### Materials

CBD reference material (purity: 99.75%) was purchased from Cayman Chemical (Ann Arbor, MI). An additional 52 standards that were used for identity confirmation were purchased from Advanced ChemBlock Inc (Hayward, CA), Aladdin Scientific (Riverside, CA), Alfa Chemicals (Binfield, England), Ambeed (Buffalo Grove, IL), Apollo Scientific (Denton, England), Beantown Chemical Corporation (Hudson, NH), Sigma-Aldrich (St. Louis, MO), TCI America (Philadelphia, PA), and Thermo Scientific (Waltham, MA). Additional file 1 shows a comprehensive list of these standards and the associated vendors. Hexane was purchased from Sigma-Aldrich (St. Louis, MO). The alkane retention index standard used to calculate retention indices was purchased from Restek (Bellefonte, PA). Helium, a 95% methane/5% ammonium gas mixture, and nitrogen were purchased from Middlesex Gases and Technology (Everett, MA).

### Pyrolysis – GC × GC – MS analysis

A JMS-T2000GC AccuTOF™ GC-Alpha gas chromatograph – high resolution-mass spectrometer (GC – HRMS) (JEOL USA, Inc., Peabody, MA) equipped with a PY-3030D Micro-Furnace Pyrolyzer and AS-2020E Auto-Shot Sampler (Frontier Lab, Koriyama, Japan) was used for these studies. This pyrolyzer system was attached directly to the split/splitless inlet to allow the direct injection of the pyrolysis products into the GC system for analysis. Additionally, an INSIGHT-Thermal Modulator (SepSolve Analytical, Waterloo, Canada) was used to enable GC × GC – HRMS analysis. A schematic illustrating the instrumental set up can be found in Additional file 2.

For each experiment, approximately 0.25 mg of CBD in powder form was placed in an Eco-cup LF long 8 mm pyrolysis cup (Frontier Lab, Koriyama, Japan). The samples were then pyrolyzed at 400 °C, 500 °C, 600 °C, 700 °C, and 800 °C by dropping the pyrolysis cup into the heated furnace for 30 s before injection (single-shot mode) into the GC × GC – HRMS system.

For the GC × GC – HRMS measurements, a ZB-5MS Plus column (30 m, 0.25 mm, 0.25 μm) (Phenomenex, Torrance, CA) was used as the first-dimension column, and a ZB-35 column (1.5 m, 0.18 mm, 0.18 μm) (Phenomenex, Torrance, CA) was used as the second-dimension column. The GC inlet temperature was set to 300 °C, the split ratio was set to 100:1, and the helium carrier gas flow rate was set to 1.0 mL/min for each measurement. For the GC temperature program, the oven was ramped from 50–280 °C at a rate of 3 °C/min and then held at 280 °C for 11.4 mins for a total run time of 90 mins. The GC × GC thermal modulator hot jet program had an initial temperature of 170 °C that was held for 2 mins before increasing at a rate of 3 °C/min to 395 °C and held for 13 mins. For the cold jet program, the initial nitrogen gas flowrate was set to 20 L/min for 2 mins and was then ramped down at a rate of 0.2 L/min^2^ to 4.50 L/min and held for 10.4 mins. A GC × GC thermal modulation period of 6 s was used for all measurements.

The HRMS was set up to use electron ionization (EI) positive ion mode for database matching, and chemical ionization (CI) positive ion mode for determining accurate molecular mass information. For the EI measurements, the system parameters were set to an ionization voltage of 70 eV, a detector voltage of 2200 V, a mass range of *m/z* 35–600, and a spectrum recording interval of 25 Hz (0.04 s). For CI measurements, the system parameters were set to an ionization voltage of 200 eV with a CI gas mixture of 95% methane/5% ammonium, a detector voltage of 2350 V, a mass range of *m/z* 60–800, and a spectrum recording interval of 25 Hz (0.04 s). A single-point drift compensation was performed for each file using column bleed peaks C_5_H_15_O_3_Si_3_^+^ (*m/z* 207.0324) and C_7_H_21_O_4_Si_4_^+^ (*m/z* 281.0511) to ensure the mass accuracy of the high-resolution EI and CI data. Three replicates at each pyrolysis temperature were analyzed using EI mode for a total of 15 measurements, and one replicate was collected for each pyrolysis temperature using CI mode for a total of 5 measurements.

### Data processing and tentative compound identifications

The JEOL msFineAnalysis AI qualitative analysis software (JEOL, Tokyo, Japan) was used for all GC × GC – HRMS data analysis. This software combines all of the information in the EI accurate mass data, the CI accurate mass data, and the alkane retention index standard data to generate a qualitative analysis report listing the proposed identities of the analytes. More specifically, this software automatically detects and links the chromatographic peaks in the EI and CI data; uses the EI mass spectra for database matching and retention index matching; integrates the CI accurate mass data and isotope ratio information for elemental composition determination of the analyte; and finally, conducts accurate mass scoring for the observed fragments to create the analysis report. Additionally, the msFineAnalysis AI software utilizes the NIST 2023 database (National Institute of Standards and Technology, Gaithersburg, MD) and the Wiley 12 database (Hoboken, NJ) for EI mass spectral matching. However, when a compound cannot be identified using these databases, then the software relies on the msFineAI library (JEOL, Tokyo, Japan) to propose possible matches. A more thorough discussion of the msFineAnalysis AI software and accompanying msFine AI database is discussed elsewhere (Ubukata et al. 2020; Cody et al. 2022; Kubo et al. 2023; Kubota et al. 2025). However, it should be noted that though the software provided a suggested top-match for each peak in the chromatogram, the data presented in the qualitative analysis report was manually reviewed to ensure the accuracy of the software. In cases where the analyst disagreed with the top-match (i.e., the second, third, or even tenth match appeared to be more plausible when considered alongside the data presented in the qualitative analysis report), the manually selected match would be set as the new “top-match” and noted as the tentative identity of the peak. Thus, while the software provided potential compound identities, the final determination was made by the analyst.

### Compound identity confirmation

Authentic standards were analyzed using 1D GC whenever possible to confirm the identities of the CBD cracking products tentatively identified using the msFineAnalysis AI software. For compounds eluting before 5.60 min, 5 μL of the standard was pipetted into a 20 mL scintillation vial (Fisher Scientific, Waltham, MA) for headspace sampling. For compounds eluting after 5.6 min, the standard solutions were prepared in hexane at concentrations of 10 ppm or 100 ppm. An alkane retention index standard was also analyzed so that the retention index ladder could be used to calculate and confirm retention indices.

In this work, the GC × GC data was collected in one dimension and then remapped into two dimensions using the GC × GC modulation period (6 s in this case). As a result, when a retention index (RI) standard is used to determine the RI value for a compound, these numbers are directly relatable between 1D and 2D measurements. Notably, these index values denote the retention behavior of the compounds of interest according to a uniform scale that is experimentally determined using a mixture of closely related standard compounds, in this case, *n*-alkane hydrocarbons (Zellner et al. 2008). As a result, the RI standard can be used to correlate analyte retention behavior among different GC columns. Consequently, it is critical that an RI standard mixture is measured when comparing GC and GC × GC methods, in order for this correlation to be valid for the sample measurements. In addition to using the RI values calculated for the authentic standards and unknowns to support compound identification, a comparison of the EI fragmentation patterns for the standard and unknown was used to further confirm each compound identity.

An Agilent 8890/5977 GC–MS (Agilent, Santa Clara, CA) equipped with a GERSTEL multipurpose sampler (MPS) (GERSTEL, Linthicum, MD) was used to analyze the liquid and headspace standards. A ZB-5MS Plus column (30 m, 0.25 mm, 0.25 μm) (Phenomenex, Torrance, CA) was used for all measurements. The GC inlet temperature was set to 300 °C, the split ratio was set to 3:1 for liquid samples and splitless for headspace samples, and the helium carrier gas flow rate was set to 1.0 mL/min for each analysis. For the GC temperature program, the oven was ramped from 50–140 °C at a rate of 3 °C/min for a total run time of 30 mins. The MS was used in EI positive ion mode with an ionization energy of 70 eV and a mass range of *m/z* 35 to 600. A 3.25-min solvent delay was used for liquid injections, and no solvent delay was used for manual (headspace) injections. The MPS was used to inject 1 μL of the 10 ppm or 100 ppm solution, and 5 μL was manually injected for the headspace samples.

## Results

**Fig. 2:**
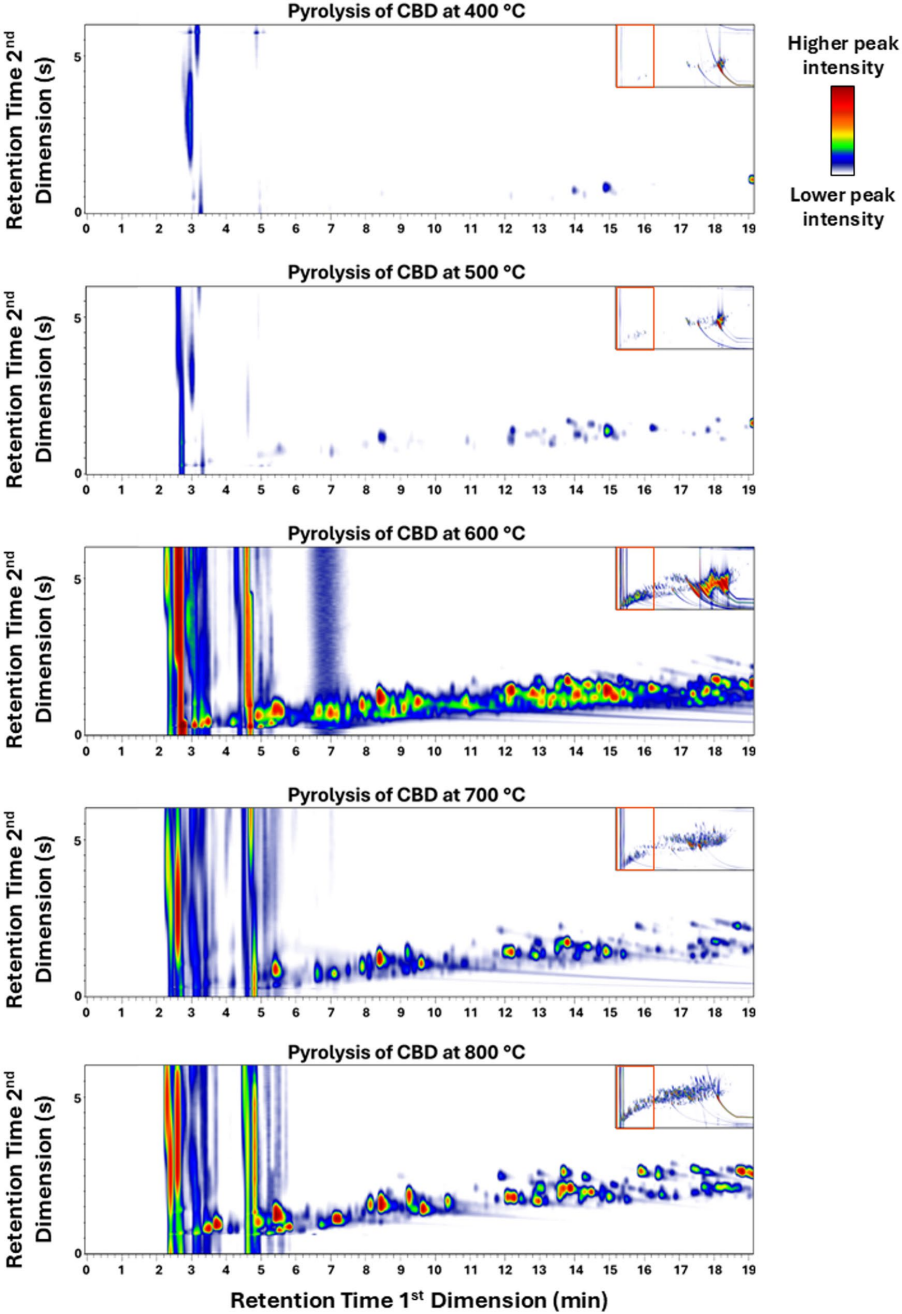
Representative GC × GC chromatograms generated using GC Image (Lincoln, NE) for the products of CBD pyrolysis at 400 °C, 500 °C, 600 °C, 700 °C, and 800 °C under anaerobic conditions. Each panel shows the elution profile for the first 19 min of a 90-min GC analysis. The chromatogram for the entire 90-min analysis is shown in the inset. The color red indicates a higher intensity peak, and the color blue indicates a lower intensity peak

Figure 2 shows representative GC × GC chromatograms of the products of CBD pyrolysis as a function of increasing temperature under anaerobic conditions. In each of the panels, the results obtained at 400 °C, 500 °C, 600 °C, 700 °C, and 800 °C show the elution profile spanning the first 19 mins, while the full chromatogram appears in the inset. Preliminary analysis of the chromatograms revealed a number of trends. One is that their complexity increased dramatically with increasing temperature, thus resulting in the detection of hundreds of compounds across each 2D chromatogram. We chose here to focus on the cracking products observed within the first 19 mins of each measurement, not only because the compounds detected over this timeframe were quite similar in a number of respects, but also because the compounds detected beyond this timeframe were significantly different in structure (e.g., dominated by the presence of fused aromatic systems, for example). Furthermore, our focus in this work was the lack of studies and reports discussing early eluting CBD pyrolysis products. The pyrolysis at each temperature was performed in triplicate and of the molecules that were detected in all three of each of the replicates, 7, 33, 48, 47, and 50 compounds were observed at 400 °C, 500 °C, 600 °C, 700 °C, and 800 °C, respectively, during the first 19 mins of the GC analysis. This represents a 614% increase in the number of compounds detected from 400 °C to 800 °C in that region of the chromatogram. Overall, 86 compounds were observed during the first 19 mins, spanning the alkane (both cyclic and acyclic), alkene (both cyclic and acyclic), alkyne, substituted benzene, aromatic heterocycle, phenol, and ketone classes. Second, all of the molecules observed within the first 19 mins were of a mass lower than that of the CBD precursor (i.e., they were cracking products). Third, as described in more detail below, approximately 41% of the compounds detected (35 compounds) in the first 19 mins appeared at only a single temperature. On the other hand, there were four cracking products that appeared at all five temperatures. Multiple compounds appeared at several of the temperatures. The cracking products were initially identified using the msFineAnalysis AI software which utilized all of the available information from the EI and CI data together, including EI mass spectral fragmentation patterns, accurate masses of molecular ions and fragments, isotope ratio matching, and retention index information to determine the most likely candidate for each peak. These candidates were then confirmed by comparing their retention indices and fragmentation patterns with those of authentic standards when available. A complete list of the products eluting during the first 19 mins of the GC × GC – HRMS analysis can be found in Table 1, presented in order of increasing first dimension retention times. The findings, including the compounds identified and the temperatures at which they appeared, are described below by compound class.

**Table 1:** CBD cracking products observed in the first 19 min of the GC × GC – HRMS analysis. Products are sorted by ascending first-dimension retention times. The temperature(s) at which the indicated compounds were observed is(are) indicated by “X”. Citations for the literature supporting each compounds’ toxicological classification are listed in the “Reference” column. The numbers correspond with the references listed in Additional file 3

#	Compound Name	Reference	Formula	Average RT First Dimension (mins)	Average RT Second Dimension (s)	400 °C	500 °C	600 °C	700 °C	800 °C
1	Cyclopropane*		C_3_H_6_	2.32	4.01			X	X	X
2	*(Z)-*2-Butene^‡^*	46	C_4_H_8_	2.41	2.59			X	X	X
3	1,3-Pentadiene^†^*	58	C_5_H_8_	2.61	3.12		X	X	X	X
4	*(Z)-*4-Methyl-2-pentene**		C_6_H_12_	2.79	4.25				X	X
5	2-Methylpentane^#^*	70	C_6_H_14_	2.81	4.30	X	X	X		
6	*n*-Hexane^‡^*	65, 69	C_6_H_14_	2.97	2.34	X	X	X	X	X
7	*(Z)-*3-Methyl-2-pentene*		C_6_H_12_	3.13	1.08			X	X	
8	2,3-Dimethyl-1,3-butadiene*		C_6_H_10_	3.17	2.51					X
9	Methylcyclopentane^‡^*	44	C_6_H_12_	3.23	3.79		X	X	X	X
10	2-Methyl-1,3-cyclopentadiene**		C_6_H_8_	3.41	2.26		X	X	X	X
11	1-Methylcyclopentene*		C_6_H_10_	3.51	2.05			X		
12	Benzene^†‡^*	49, 68	C_6_H_6_	3.66	1.52				X	X
13	1,3-Cyclohexadiene*		C_6_H_8_	3.71	2.36			X	X	X
14	*(Z)-*3,4-Dimethyl-2-pentene*		C_7_H_14_	3.91	0.34				X	
15	*(Z)-*3-Methyl-3-hexene*		C_7_H_14_	4.07	1.04			X	X	X
16	3,4-Heptadiene**		C_7_H_12_	4.27	0.46			X	X	X
17	3-Ethenylcyclopentene**		C_7_H_10_	4.93	0.88			X	X	X
18	2-Methylcyclohexa-1,3-diene**		C_7_H_10_	5.17	0.83					X
19	Toluene^†‡^*	49	C_7_H_8_	5.41	0.94				X	X
20	1-Methylcyclohexa-1,3-diene*		C_7_H_10_	5.57	0.64		X	X	X	X
21	4-Methyl-1,4(*E*)-heptadiene**		C_8_H_14_	5.98	0.42			X		
22	1,3-Cycloheptadiene*		C_7_H_10_	6.64	0.69					X
23	5,6-Dimethyl-1,3-cyclohexadiene**		C_8_H_12_	6.68	0.79		X	X	X	
24	1,4-Dimethylcyclohexene**		C_8_H_14_	7.04	0.73		X	X		
25	3-Methylidenecyclohexa-1,4-diene**		C_7_H_8_	7.11	0.87					X
26	1,2-Dimethyl-1,4-cyclohexadiene**		C_8_H_12_	7.51	0.89				X	
27	1,5-Dimethyl-1,4-cyclohexadiene**		C_8_H_12_	7.71	0.90				X	
28	2,3-Dimethylcyclohexa-1,3-diene^✕**^	86	C_8_H_12_	7.90	0.98		X	X	X	
29	Ethylbenzene^†^*	51	C_8_H_10_	8.11	1.27				X	X
30	1,2-Dimethylcyclohexene**		C_8_H_14_	8.34	0.63			X		
31	*p*-Xylene^†‡^*	49	C_8_H_10_	8.41	1.19	X	X	X	X	X
32	Unidentified		-	8.81	0.87		X			
33	3-Methylcyclopenta-2,4-dien-1-one**		C_6_H_6_O	9.01	2.21					X
34	4-Methylideneocta-2,5-diene**		C_9_H_14_	9.08	0.91			X		
35	*m*-Xylene^†‡^*	49	C_8_H_10_	9.21	1.48				X	
36	Styrene^#^*	71	C_8_H_8_	9.21	1.63					X
37	Unidentified		C_8_H_12_	9.24	1.03		X			
38	*o*-Xylene^†‡^*	49	C_8_H_10_	9.57	1.10		X	X	X	X
39	1-Methylene-3-(1-methylethylidene)-cyclopentane**		C_9_H_14_	11.11	1.09		X	X		
40	1-Prop-1-ynylcyclopentene**		C_8_H_10_	11.11	1.85					X
41	3,4-Bis(methylene)-cyclopentanone**		C_7_H_8_O	11.81	2.23					X
42	1-Ethyl-4-methylbenzene^†#^*	54, 64	C_9_H_12_	11.94	1.39			X	X	X
43	Verbenene**		C_10_H_14_	12.14	1.39		X			
44	1-Ethyl-3-methylbenzene^#^*	73	C_9_H_12_	12.21	1.50				X	X
45	2-Ethyl-3-methylcyclohexa-1,3-diene**		C_9_H_14_	12.68	1.32		X			
46	1,3,5-Trimethylbenzene ^‡#^*	63, 72	C_9_H_12_	12.72	1.36		X	X	X	X
47	1-Ethyl-2-methylbenzene^#^*	73	C_9_H_12_	12.74	1.45					X
48	Phenol^†^*	49	C_6_H_6_O	12.91	2.33					X
49	α-Methylstyrene^†#^*	62	C_9_H_10_	12.95	1.77			X	X	X
50	Unidentified		C_10_H_16_	13.11	1.01			X		
51	β-Myrcene^✕^*	74	C_10_H_16_	13.31	1.00		X	X		
52	1,2,4-Trimethylbenzene^†‡#^*	56, 57, 59, 61, 66	C_9_H_12_	13.31	1.46		X	X	X	
53	1-Ethenyl-2-methylbenzene^†‡^*	60	C_9_H_10_	13.34	1.89					X
54	1-Ethenyl-3-methylbenzene^†‡^*	47, 60	C_9_H_10_	13.54	1.85					X
55	Benzofuran^†^*	48	C_8_H_6_O	13.57	2.33				X	X
56	1-Ethenyl-4-methylbenzene^†‡^*	47, 60	C_9_H_10_	13.81	1.77		X	X	X	X
57	1,2,3-Trimethylbenzene^†‡^*	53, 66, 72	C_9_H_12_	14.00	1.58		X	X	X	
58	3-Ethenyl-1,2-dimethyl-1,4-cyclohexadiene**		C_10_H_14_	14.03	1.19	X	X			
59	3,3-Dimethyl-6-methylenecyclohexene**		C_9_H_14_	14.18	1.22			X		
60	6-Ethyl-2,6-dimethyl-1,3-cyclohexadiene**		C_10_H_16_	14.31	1.05	X	X			
61	1-Propenylbenzene^†#^*	50, 60	C_9_H_10_	14.35	1.64			X	X	X
62	1-Methyl-4-(1-methylethyl)−1,3-cyclohexadiene^†^**	45, 67	C_10_H_16_	14.64	1.35		X			
63	*o*-Cymene^✕^*	78	C_10_H_14_	14.68	1.38			X		
64	*m*-Cymene^✕^*	79	C_10_H_14_	14.87	1.42	X	X	X	X	X
65	*D*-Limonene^✕^*	75, 76, 77	C_10_H_16_	15.11	1.18			X		
66	Indane^✕^*	88	C_9_H_10_	15.14	1.89				X	X
67	5-(1-Methylpropylidene)−1,3-cyclopentadiene**		C_9_H_12_	15.31	1.73			X		
68	*p*-Cymene^✕^*	80	C_10_H_14_	15.43	1.42		X	X	X	X
69	1-Propynylbenzene*		C_9_H_8_	15.82	2.30			X	X	X
70	1-Methyl-2-propylbenzene*		C_10_H_14_	16.11	1.58				X	X
71	(2-Methylprop-1-enyl)-cyclohexa-1,5-diene**		C_10_H_14_	16.21	1.55		X			
72	1-Ethynyl-4-methylbenzene*		C_9_H_8_	16.31	2.23				X	X
73	1,2-Diethylbenzene^‡^*	52, 53	C_10_H_14_	16.44	1.54			X	X	X
74	Unidentified		-	16.74	1.01			X		
75	Acetophenone^✕^*	90, 91	C_8_H_8_O	17.01	2.88					X
76	4-Methyl-1-(1-methylethenyl)-cyclohexene^✕**^	89	C_10_H_16_	17.11	1.36		X	X		
77	1-Ethyl-2,4-dimethylbenzene*		C_10_H_14_	17.34	1.71					X
78	3-Methylphenol†*	49	C_7_H_8_O	17.41	2.39				X	X
79	3-Methyl-6-(1-methylethylidene)-cyclohexene^✕**^	81	C_10_H_16_	17.51	1.33		X	X	X	
80	1-Methyl-3-(1-methylethenyl)-benzene^✕^*	85	C_10_H_12_	17.61	1.85				X	X
81	1-Methyl-4-(1-methylethylidene)-cyclohexene^✕^*	81	C_10_H_16_	17.88	1.41		X	X	X	
82	1-Methyl-4-(1-methylethenyl)-benzene^✕^*	83	C_10_H_12_	18.19	1.73		X	X	X	X
83	Unidentified		C_10_H_16_	18.26	1.21		X	X		
84	2-Ethenyl-1,4-dimethylbenzene*		C_10_H_12_	18.51	1.86					X
85	2-Methylbenzofuran*		C_9_H_8_O	18.54	2.29			X	X	X
86	1,3,8-*p*-Menthatriene^✕**^	84, 87	C_10_H_14_	18.94	1.50	X	X	X	X	

*Identities were confirmed using authentic standards. **Identified based on fragmentation pattern and retention index matching against the NIST Library, Wiley database, or msFineAnalysis AI Library. ^†^Reported to exhibit genotoxic properties. ^‡^Reported to exhibit neurotoxic properties. ^#^Reported to exhibit other negative health effects. ^✕^Reported to have beneficial health properties

### Alkanes

Four alkanes were identified. Two are acyclic, namely the constitutional isomers *n*-hexane and 2-methylpentane. While the former appeared at all five temperatures, the latter was observed at the three temperatures 400 °C, 500 °C, and 600 °C. The other two alkanes were cyclopropane (detected at 600 °C, 700 °C, and 800 °C), and methylcyclopentane (detected at all measurement temperatures except 400 °C).

### Alkenes (acyclic)

Eleven acyclic alkenes were identified. Five were monounsaturated, namely: *cis*−2-butene, the isomeric pair (*Z*)−4-methyl-2-pentene and (*Z*)−3-methyl-2-pentene, and a second isomeric pair, (*Z*)−3,4-dimethyl-2-pentene and (*Z*)−3-methyl-3-hexene. Five were dienes, namely: (*Z*)−1,3-pentadiene, 2,3-dimethyl-1,3-butadiene, 3,4-heptadiene, 4-methyl-1,4(E)-heptadiene, and 4-methylideneocta-2,5-diene. One was a triene, β-myrcene. Some acyclic alkenes were detected at only one temperature (e.g., 4-methyl-1,4-heptadiene at 600 °C). Others were detected at multiple temperatures (e.g., β-myrcene at 500 °C and 600 °C).

### Alkenes (cyclic)

The largest number of pyrolysis products fell within this class, with a total of 28. Monounsaturated, diene, and trienes were all observed. The simplest of the monounsaturated cycloalkenes and the temperatures at which they were observed were 1-methylcyclopentene (600 °C), and the isomers 1,4-dimethylcyclohexene and 1,2-dimethylcyclohexene (detected at 500 °C – 600 °C and 600 °C, respectively). Next were several dienes, with the simplest of these being unsubstituted 1,3-cyclohexadiene (600 °C – 800 °C) and 1,3-cycloheptadiene (800 °C). In most of the remaining dienes, both double bonds were endocyclic (e.g., monosubstituted ring compounds such as 2-methyl-1,3-cyclopentadiene (500 °C – 800 °C) and the isomers 1- and 2-methylcyclohexa-1,3-diene (500°C - 800 °C and 800 °C respectively); disubstituted isomers 5,6-diemthyl-1,3-cyclohexadiene (500 °C – 700 °C), 1,2-dimethyl-1,4-cyclohexadiene (700 °C), 1,5-dimethyl-1,4-cyclohexadiene (700 °C), and 2,3-dimethylcyclohexa-1,3-diene (500 °C – 700 °C); 2-ethyl-3-methylcyclohexa-1,3-diene (500 °C), 6-ethyl-2,6-dimethyl-1,3-cylohexadiene (400 °C – 500 °C), and 1-methyl-4(1-methylethyl)−1,3-cyclohexadiene (500 °C)). The remaining dienes exhibited at least one exocyclic double bond. There was one case where both double bonds were exocyclic (1-methylene-3-(1-methylethylidene)-cyclopentane (500 °C—600 °C)), and seven compounds in which one double bond was exocyclic and the other endocylic. These were 3-ethenyl-1-cyclopentene (600–800 °C), 3,3-dimethyl-6-methlenecyclohexene (600 °C), 4-methyl-1-(1-methylethenyl)-cyclohexene (500–600 °C), 3-methyl-6-(1-methylethylidene)-cyclohexene (500 °C – 700 °C), 1-methyl-4-(1-methylethylidene)-cyclohexene (500 °C – 700 °C), and two naturally occurring terpenes, the bicyclic verbenene (500 °C) and *D*-limonene (600 °C). The remaining cyclic alkenes were all trienes with one exocyclic and two endocyclic double bonds. These compounds were 3-methylidenecyclohexa-1,4-diene (800 °C), 3-ethenyl-1,2-dimethyl-1,4-cyclohexadiene (400 °C – 500 °C), 5-(1-methylpropylidene)−1,3-cyclopentadiene (600 °C), (2-methylprop-1-enyl)-cyclohexa-1,5-diene (500 °C), and the terpenoid natural product 1,3,8-*p*-menthatriene (400 °C – 700 °C).

### Alkynes

One aliphatic alkyne was consistently detected in all replicates that were pyrolyzed at 800 °C —1-prop-1-ynylcyclopentene.

### Benzene and various mono-, di-, and trisubstituted derivatives

A wide variety of substituted benzenes were observed. Unsubstituted benzene was detected at 700 °C – 800 °C. Five compounds were monosubstituted, namely toluene and ethylbenzene, both observed at 700 °C – 800 °C, styrene (800 °C), 1-propenylbenzene (600 °C – 800 °C), and α-methylstyrene (600 °C – 800 °C). Several groups of isomeric disubstituted benzenes were identified. These included *o-*, *m-,* and *p*-xylenes (observed at 500 °C – 800 °C for *o*-xylene, 700 °C for *m*-xylene, and 400 °C – 800 °C for *p*-xylene); *o*-, *m*-, and *p*-ethyl methyl benzenes (observed at 800 °C, 700 °C – 800 °C, and 600 °C – 800 °C, respectively); *o*-, *m*-, and *p*-ethenyl methyl benzenes (observed at 800 °C, 800 °C, and 500 °C – 800 °C, respectively); *o*-, *m*-, and *p*-cymenes (at 600 °C, 400 °C – 800 °C, and 500 °C – 800 °C, respectively); *m*- and *p*-methyl-(1-methylethenyl) benzenes (detected at 700 °C – 800 °C and 500 °C – 800 °C, respectively); the two C_10_H_14_ isomers 1-methyl-2-*n*-propylbenzene (700 °C – 800 °C) and 1,2-diethylbenzene (600 °C – 800 °C); and the two isomeric alkynes 1-propynylbenzene (600 °C – 800 °C) and 1-ethynyl-4-methylbenzene (700 °C – 800 °C). Five trisubstituted benzenes were observed: three isomeric trimethylbenzenes (1,2,3-, 1,2,4-, and 1,3,5- trimethylbenzenes at 500 °C – 700 °C, 500 °C – 700 °C, and 500 °C – 800 °C, respectively); 1-ethyl-2,4-dimethylbenzene (800 °C); and 2-ethenyl-1,4-dimethylbenzene (800 °C).

### Bicyclic aromatic hydrocarbons

At the temperatures 700 °C and 800 °C, the fused C_9_H_10_ bicyclic hydrocarbon indane was observed.

### Bicyclic heteroaromatic hydrocarbons

Two structurally related compounds representing this class were identified—benzofuran (700 °C – 800 °C) and 2-methylbenzofuran (600 °C – 800 °C).

### Phenols

Two phenols were identified—phenol itself (800 °C), and 3-methylphenol (700 °C – 800 °C).

### Ketones

Three ketones were detected, all cyclic, and all were observed only at 800 °C. These were 3-methylcyclopenta-2,4-diene-1-one; 3,4-bis(methylene)-cyclopentanone; and acetophenone.

The temperatures at which the aforementioned products were formed are listed in Table 1. Three compounds, *n*-hexane, *p*-xylene, and *m*-cymene were formed at all five temperatures. Ten compounds appeared at four of the temperatures: 1,3,8-*p*-menthatriene was detected from 400–700 °C, and the following nine compounds appeared from 500–800 °C: 1,3-pentadiene, methylcyclopentane, 2-methyl-1,3-cyclopentadiene, 1-methylcyclohexa-1,3-diene, *o*-xylene, 1,3,5-trimethylbenzene, 1-ethenyl-4-methylbenzene, *p*-cymene, and 1-methyl-4-(1-methylethenyl)benzene. Other compounds, as shown in Table 1, appeared at three, two, or only one of the measurement temperatures.

## Discussion

It has been shown previously that when CBD (C_21_H_30_O_2_) is pyrolyzed, multiple products with molecular weights both higher and lower than that of CBD are formed. To date, 29 pyrolysis products have been reported, ranging in carbon number from 10 to 21. However, it was acknowledged in several of these earlier studies that the full range of detected molecules was not determined, in part because of the challenge of compound coelution that made structure identification of molecules by 1D GC–MS or liquid chromatography-MS impossible (Tjeerdema 1987; Küppers et al. 1975a, 1975b; Küppers et al. 1973; Luteyn et al. 1978; Mikeš and Waser 1971; Salemink and Nahas 1976; Spronck and Lousberg 1977; Spronck et al. 1978; Spronck and Salemink 1978; Czégény et al. 2021). Furthermore, emphasis was placed on identification of compounds with carbon numbers ≥ 10. In this work, the issues associated with compound co-elution were addressed by using GC × GC. This technique, coupled with HRMS, EI-MS fragmentation pattern matching, and retention index information, resulted in the identification of 81 compounds, from a total of 86 that were detected (see Table 1). The structural assignments for 52 of these compounds were further confirmed through comparisons with authentic standards. Of the identified compounds, only 3 were previously reported as CBD pyrolysis products, namely 1-methyl-4-(1-methylethenyl)-benzene (*p*-cymenene), *p*-cymene, and 1,3,8-*p*-methatriene (Czégény et al. 2021). While the full GC × GC analysis method spanned 90 min, it was observed that the chromatogram exhibited three discrete regions comprising different types of compounds. The compounds reported here, which eluted within the first 19 min, were cracking products containing 3 to 10 carbons, and were primarily aliphatic or substituted benzenes. The regions between 19–54 min and 54–90 min were dominated by naphthalenes and fused multicyclic ring scaffolds including cannabinoids and related compounds, respectively (data not shown). These findings will be the subject of a future report.

**Fig. 3:**
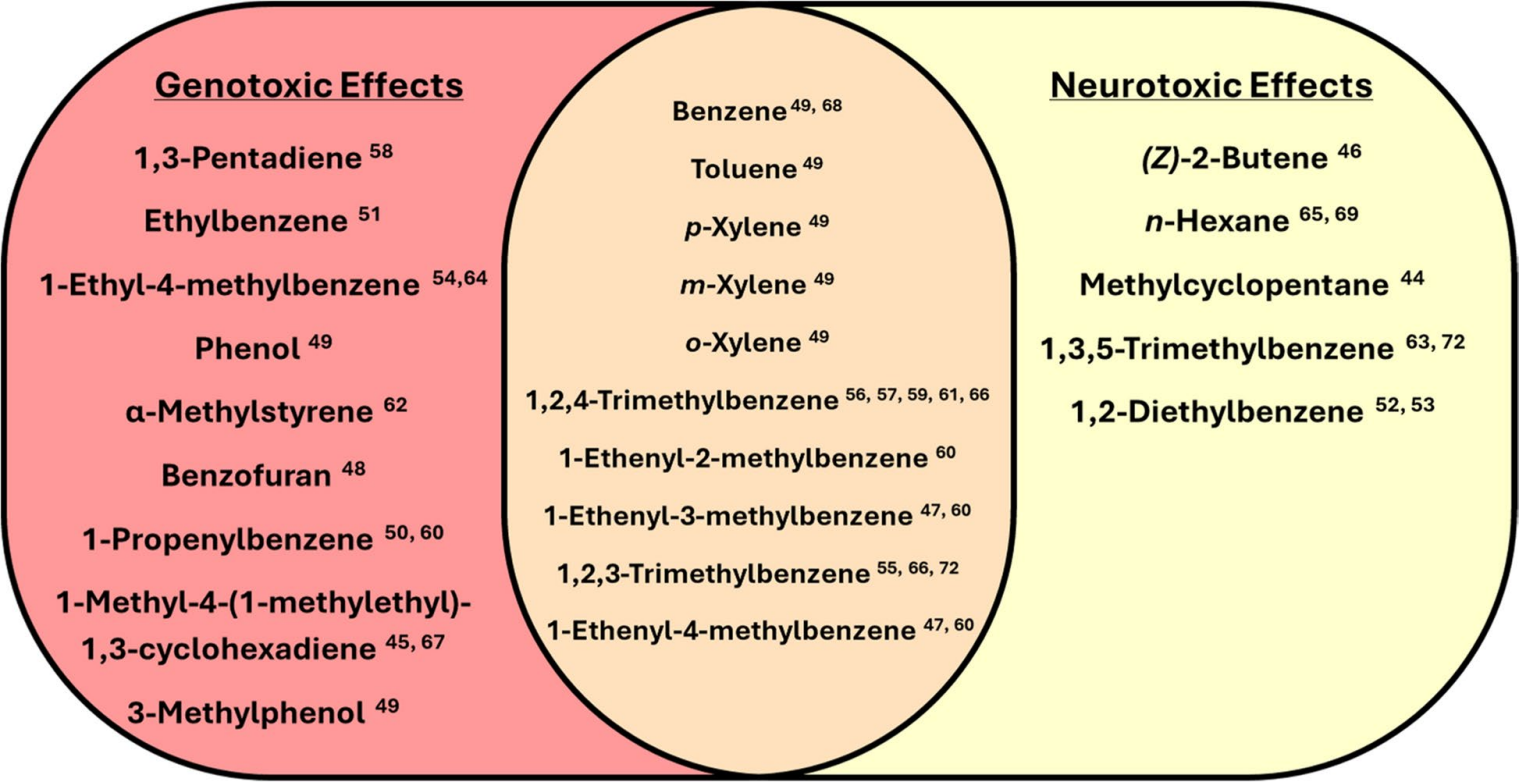
CBD cracking products identified in this study that are reported to exhibit genotoxic effects (left panel, in red), neurotoxic effects (right panel, in yellow), or both (middle panel, in orange). Reference to literature supporting the toxicological classifications are indicated by the superscripted number following each compound name. The numbers correspond with the references listed in Additional file 3

A number of the detected compounds are known to have adverse health impacts. Human exposure analysis, animal models, and biological assays have demonstrated the genotoxicity and neurotoxicity of several of the observed pyrolysis products (Developmental toxicity of two trimethylbenzene isomers 2005; Agency 2009; Baldissera et al. 2017; Bingham et al. 2021; Cancer IAfRo. 1994, 1996; Dean 1978; Gagnaire and Langlais 2005; Henderson et al. 2007; Hoang et al. 2023, 2021; Janik-Spiechowicz and Wyszyńska 1998; Janik-Spiechowicz et al. 1997; Korsak et al. 2000, 1995; Liewen and Marth 1985; Maltoni et al. 1997; Norppa and Vainio 1983; Norseth et al. 1991; Program 2007; Saillenfait et al. 2005; Świercz et al. 2000; AfTSaD 2025; Wiaderna et al. 1998; Wojtunik-Kulesza 2022; Yardley-Jones et al. 1991; Zhang et al. 2023). Other effects, including respiratory impacts, skin and eye irritation, and low birthrate effects have also been reported in mammalian models (Gagnaire and Langlais 2005; Janik-Spiechowicz and Wyszyńska 1998; Korsak et al. 2000, 1995; Maltoni et al. 1997; Norppa and Vainio 1983; Norseth et al. 1991; Program 2007; Saillenfait et al. 2005; Świercz et al. 2000; Wiaderna et al. 1998; Galvin and Bond 1999; Gibbs and Mulligan 1997; Korsak and Rydzynski 1996; Roberts et al. 2017). Those found to be neuro- and/or genotoxic are shown in Fig. 3. For example, benzene and several of its substituted derivatives, such as toluene, ethylbenzene, the xylenes, and the styrenes, as well as phenol and the identified phenol derivatives, have been shown to be cancer causing in human and animal studies (Cancer IAfRo. 1994; Dean 1978; Henderson et al. 2007; Norppa and Vainio 1983; Program 2007; Yardley-Jones et al. 1991). On the other hand, the aliphatic compounds such as *n*-hexane, methylcyclopentane, and 2-butene, have been shown to be neurotoxic in humans and in animal models (Agency 2009; Bingham et al. 2021; AfTSaD 2025; Zhang et al. 2023). Some compounds, including the trimethylbenzenes, exhibit both geno- and neurotoxic effects as described in studies observing human and animal exposure impacts (Janik-Spiechowicz et al. 1997; Korsak et al. 2000, 1995; Maltoni et al. 1997; Norseth et al. 1991; Wiaderna et al. 1998).

Interestingly, pyrolysis of CBD also produced a number of naturally occurring compounds. These included *D*-limonene and β-myrcene, common plant-derived natural products found in fruits, essential oils and in *Cannabis* itself (1). Both *D*-limonene and β-myrcene are used commercially in the flavor and fragrance industries and may contribute to the organoleptic properties of *Cannabis* cigarettes (Surendran et al. 2021; Hard and Whysner 1994; AlSaffar et al. 2022; Sun 2007). The three cymenes are also known plant natural products and are reported to exhibit antibacterial, antimicrobial, and anti-inflammatory activity in animal studies and biological assays (Andrade et al. 2014; Marques et al. 2019; Marchese et al. 2017). Other compounds, all natural products, are reported to have antimicrobial, antibacterial, anti-inflammatory, and antioxidant activity in animal models and studies of cellular systems. These include 1-methyl-3-(1-methylethenyl)-benzene, 1-methyl-4-(1-methylethenyl)-benzene, 1-methyl-4-(1-methylethylidene)-cyclohexene, 1,3,8-*p*-methatriene, 2,3-dimethyl-cyclohexa-1,3-diene, 3-methyl-6-(1-methylethylidene)-cyclohexene, 4-methyl-1-(1-methhylethenyl)-cyclohexene, acetophenone, and indane (Alali and Al-Lafi 2003; Aydin et al. 2013; Baginska et al. 2023; Di Napoli et al. 2023; Flach et al. 2002; Hashemi et al. 2013; Nasir Shah et al. 2023; Prasher and Sharma 2021; Shafaghat 2010; Zubkov and Kouznetsov 2023; Api et al. 2018). There were 38 compounds whose impacts on human and animal health have not been reported. Examples are cyclopropane, multiple dienes and trienes, the alkyne, and verbenene (which also happens to be a naturally occurring compound) among others (see Table 1).

It should be acknowledged that many of the health effects previously discussed are dose-dependent. Thus, the amount of the compound to which a user is exposed, and how often that exposure occurs, will determine whether there are health impacts on *Cannabis* smokers. While this work was primarily qualitative, a rough estimate of the dominant products can be inferred from examination of the GC peak areas for the identified compounds. Using 800 °C as a case in point (i.e.,the temperature at which the greatest number of compounds are formed) the nine most dominant peaks are *p*-xylene, 3-methylidenecyclohexa-1,4-diene, benzene, *o*-xylene, 2-methylbenzofuran, 1-ethenyl-4-methyl-benzene, styrene, 1-ethyl-3-methyl-benzene, and 1-ethenyl-3-methyl-benzene. Most of these peaks represent aromatic hydrocarbons, many of which were previously highlighted as being associated with adverse health effects. Nevertheless, a quantitative analysis would need to be performed to determine which compounds are at the highest concentrations at each temperature.

When considering the relative proportion of compounds formed at each temperature, additional consideration should be made for the pyrolysis conditions under which the experiment is performed. Previous work has noted that when the results of CBD pyrolysis products anaerobic vs aerobic conditions are compared, it is the relative ratios of the products formed, rather than the identities of the products themselves, that differ (Kuppers F et al. 1975a; Küppers F et al. 1975b; Salemink CA et al. 1976; Spronck H et al. 1977). The present experiments were conducted under anaerobic conditions to best reflect the conditions of the “pyrolysis zone” of a burning cigarette. It should be noted however, that previous work reporting differences in the ratios of products as a function of the presence or absence of oxygen were focused on larger pyrolysis products, and not the lower molecular weight, early eluting compounds that are the subject of the present study. Thus, it is still of interest to investigate the extent to which the pyrolysis product profiles differ when experiments are performed under aerobic conditions, with a focus on low molecular weight products. Such an investigation will be the subject of future work.

A subset of the compounds observed have been detected in *Cannabis* smoke and its condensate. These include 1-ethenyl-3-methylbenzene, 1-ethyl-2-methylbenzene, 1-ethyl-3-methylbenzene, 1-ethyl-4-methylbenzene, 1-methyl-2-propylbenzene, 1-methyl-3-(1-methylethenyl)-benzene, 1-methyl-4-(1-methylethenyl)-benzene, 1-methyl-4-(1-methylethyl)−1,3-cyclohexadiene, 1-methyl-4-(1-methylethylidene)-cyclohexene, 1-propynylbenzene, 1,2-diethylbenzene, 1,2,3-trimethylbenzene, 1,2,4-trimethylbenzene, 1,3,5-trimethylbenzene, 1,3,8-*p*-menthatriene, 2-ethenyl-1,4-dimethylbenzene, 2-methylbenzofuran, 3-methylphenol, 3,3-dimethyl-6-methylenecyclohexene, 3,4-bis(methylene)-cyclopentanone, acetophenone, benzene, *D*-limonene, ethylbenzene, indane, *m*-xylene, *o*-cymene, *o*-xylene, *p*henol, *p*-xylene, styrene, toluene, α-methylstyrene, and β-myrcene (Moir et al. 2008; Graves et al. 2020; Sparacino et al. 1990). As these compounds are released from burning *Cannabis*, and several are not present in bulk unburned plant material, the results indicate that they may be formed by CBD pyrolysis, although the possibility that they are derived from other pyrolyzed compounds cannot yet be ruled out.

Besides its use as a recreational drug, *Cannabis* is also smoked to relieve some of the side effects associated with cancer treatment, including nausea and vomiting, pain, appetite suppression, stress, anxiety, and insomnia. However, our findings regarding the broad range of carcinogens and other harmful compounds formed from pyrolysis of the major cannabinoid CBD, and to which smokers may be chronically exposed, raise concerns about the possible additional adverse effects that may be incurred. The results presented here provide more detailed information on the identities of these compounds, and this information can inform investigations not only of the content of inhaled *Cannabis* cigarette smoke, but also, the impact of exposure in biological systems.

## Conclusions

Pyrolysis of CBD over a range of five temperatures yielded a total of 86 compounds that eluted during the first 19 min of a 90-min GC × GC – HRMS analysis. Eighty-one of these compounds were structurally assigned using mass spectral database comparisons in combination with retention indices and accurate masses. Further, the identities of 52 of these compounds were confirmed by comparison with authentic standards. Seventy-eight compounds are reported for the first time as being CBD pyrolysis products. The number of pyrolysis products increased as a function of increasing temperature. The greatest number of compounds (50 in total) were detected at 800 °C, while only 7 cracking products were detected at 400 °C. Represented compound classes included alkanes, alkenes, an alkyne, cycloalkanes, cycloalkenes, aromatic hydrocarbons, ketones, phenols, and heterocyclic compounds. Three compounds were observed at all five temperatures: *n*-hexane, *p*-xylene, and *m*-cymene, while others were observed at only a single temperature. Additionally, many of the detected molecules have been linked to adverse health effects in biological systems including cell, animal, and human models, whereas other compounds are linked to a variety of health benefits. The reported health effects are dose-dependent. Thus, future quantification experiments will be needed to determine whether the quantities of each product rise to the levels that have been found to exert biological effects. Several of the pyrolysis products have been detected in *Cannabis* smoke, indicating that CBD may serve as a precursor for these compounds. Future studies will aim to determine the extent to which CBD pyrolysis contributes to the products detected in *Cannabis* smoke.

## Supplementary Information

Additional file 1: Authentic Standards Vendor List. Commercially available standards used for compound identity confirmation, and the corresponding suppliers.

Additional file 2: Instrument Set Up. Schematic of the instrument set up used for the pyrolysis and GC × GC – HRMS analysis of CBD.

Additional file 3: References cited in Figure 1, Table 1, and Figure 3.

## Data Availability

The datasets generated in this study are available from the corresponding author upon reasonable request.

## Abbreviations

1D GC: One-dimensional gas chromatography
CBC: Cannabichromene
CBL: Cannabicyclol
CBD: Cannabidiol
CBE: Cannabielsoin
CBN: Cannabinol
CI: Chemical ionization
*C. sativa*: *Cannabis* *sativa*
EI: Electron ionization
GC: Gas chromatography
GC × GC: Two-dimensional gas chromatography
HRMS: High-resolution mass spectrometry
MPS: Multipurpose system
MS: Mass spectrometry
NIST: National Institute of Standards and Technology
U.S.: United States
Δ^9^-THC: Δ^9^-Tetrahydrocannabinol
