# Supplementary material for: Detection and identification of small-molecule cracking products formed on pyrolysis of cannabidiol part 1: C3 – C10 compounds
Source: J Cannabis Res. 2026 May 1;8:90. doi: 10.1186/s42238-026-00442-8 (PMC13445818; doi:10.1186/s42238-026-00442-8)
Supplement: Supplementary file 1 [file 42238_2026_442_MOESM2_ESM.pdf]

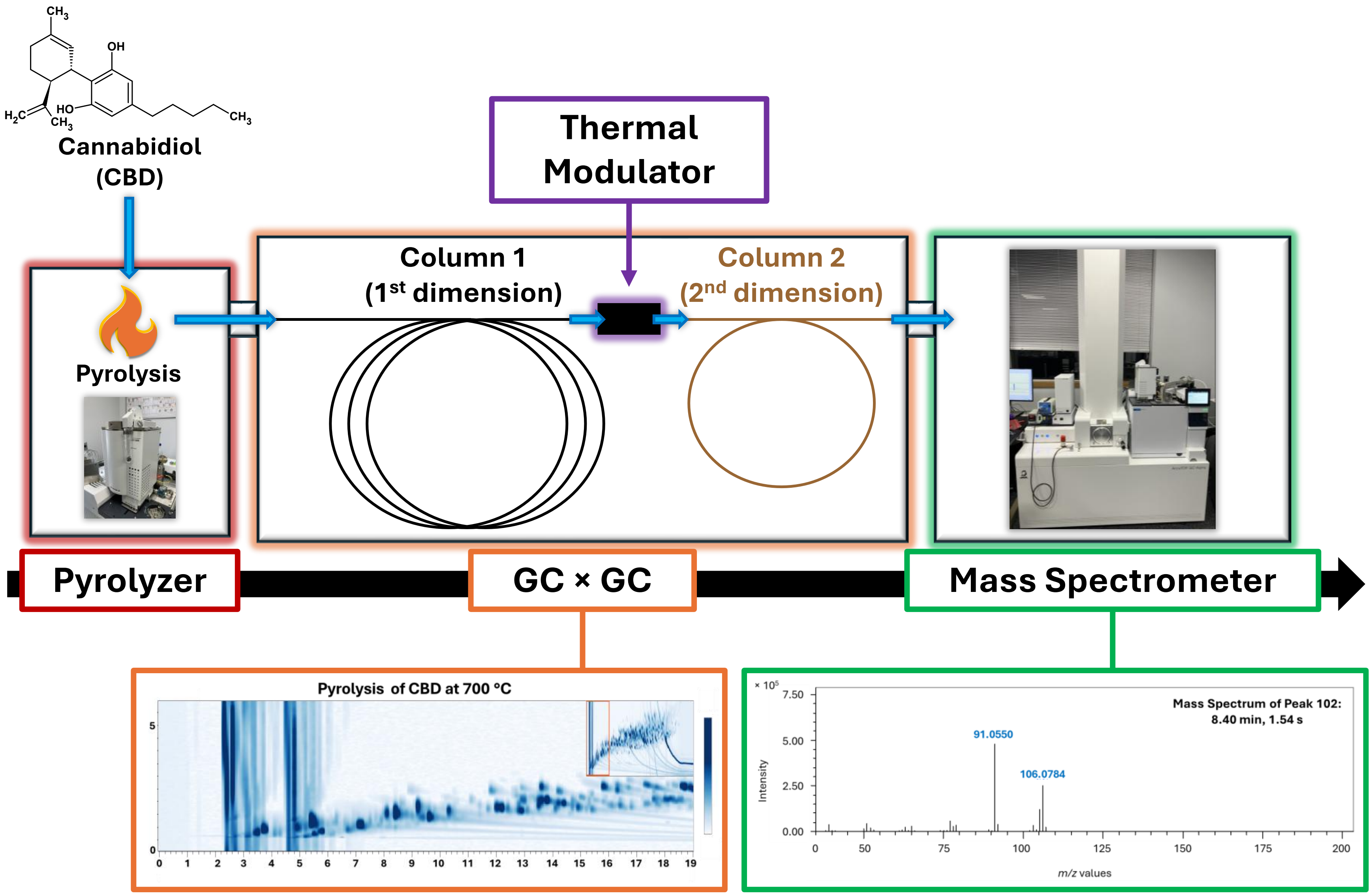

Figure S1. Schematic of the instrument set up used for the pyrolysis and GC × GC – HRMS analysis of CBD.
