# Supplementary material for: Detection and identification of small-molecule cracking products formed on pyrolysis of cannabidiol part 1: C3 – C10 compounds
Source: J Cannabis Res. 2026 May 1;8:90. doi: 10.1186/s42238-026-00442-8 (PMC13445818; doi:10.1186/s42238-026-00442-8)
Supplement: Supplementary file 2 [file 42238_2026_442_MOESM3_ESM.pdf]

List S1. References cited in Figure 1, Table 1, and Figure 3.

1. Kovalchuk I, Pellino M, Rigault P, Van Velzen R, Ebersbach J, Ashnest J, et al. The genomics of *Cannabis* and its close relatives. Annual Review of Plant Biology. 2020;71(1):713–39.
2. Sirangelo TM, Ludlow RA, Spadafora ND. Multi-omics approaches to study molecular mechanisms in *Cannabis sativa*. Plants. 2022;11(16):2182.
3. Adams R, Pease D, Clark J. Isolation of cannabinol, cannabidiol and quebrachitol from red oil of Minnesota wild hemp. Journal of The American Chemical Society. 1940;62(8):2194–6.
4. Mechoulam R, Shvo Y. Hashish—I: The structure of cannabidiol. Tetrahedron. 1963;19(12):2073–8.
5. Rao VK, Lewis-Bakker MM, Wasilewski E, Clarke HA, Kotra LP. Stereoisomers of cannabidiols and their pharmacological activities–A potentially novel direction for cannabinoids. Bioorganic & Medicinal Chemistry. 2025;117:118019.
6. Flores-Sanchez IJ, Verpoorte R. Secondary metabolism in *Cannabis*. Phytochemistry Reviews. 2008;7(3):615–39.
7. Lampe JRS, Lisa N.; Sheikh, Hassan Z. The Federal Status of Marijuana and the Policy Gap with States: U.S. Congress; 2026 IF12270:[Available from: https://www.congress.gov/crs-product/IF12270].
8. Group CICIW. Mapping Global *Cannabis* Legality 2024 [Available from: https://www.cicouncil.org.uk/map/].
9. Rock EM, Parker LA. Constituents of *Cannabis sativa*. Murillo-Rodriguez E, Pandi-Perumal SR, Monti JM, editors2020. 1–13 p.
10. ElSohly MA, Radwan MM, Gul W, Chandra S, Galal A. Phytochemistry of *Cannabis sativa L.* Kinghorn AD, Falk H, Gibbons S, Kobayashi Ji, Editors 2017. 1–36 p.
11. Radwan MM, Chandra S, Gul S, ElSohly MA. Cannabinoids, phenolics, terpenes and alkaloids of *Cannabis*. Molecules. 2021;26(9):2774.
12. Helcman M, Šmejkal K. Biological activity of *Cannabis* compounds: A modern approach to the therapy of multiple diseases. Phytochemistry Reviews. 2022;21(2):429–70.
13. Moir D, Rickert WS, Levasseur G, Larose Y, Maertens R, White P, et al. A comparison of mainstream and sidestream marijuana and tobacco cigarette smoke produced under two machine smoking conditions. Chemical Research in Toxicology. 2008;21(2):494–502.
14. Andre CM, Hausman J-F, Guerriero G. *Cannabis sativa*: The plant of the thousand and one molecules. Frontiers in Plant Science. 2016;7:19.
15. Elhendawy MA, Wanas AS, Radwan MM, Azzaz NA, Toson ES, ElSohly MA. Chemical and biological studies of *Cannabis sativa* roots. Medical *Cannabis* and Cannabinoids. 2019;1(2):104–11.
16. Huang S, Li H, Xu J, Zhou H, Seeram NP, Ma H, et al. Chemical constituents of industrial hemp roots and their anti-inflammatory activities. Journal of *Cannabis* Research. 2023;5(1):1.
17. Kornpointner C, Martinez AS, Marinovic S, Haselmair-Gosch C, Jamnik P, Schröder K, et al. Chemical composition and antioxidant potential of *Cannabis sativa L.* roots. Industrial Crops and Products. 2021;165:113422.

18. Yadav SPS, Kafle M, Ghimire NP, Shah NK, Dahal P, Pokhrel S. An overview of phytochemical constituents and pharmacological implications of *Cannabis sativa L.* Journal of Herbal Medicine. 2023;42:1–14.
19. Fehr KOB, Kalant H. Analysis of *Cannabis* smoke obtained under different combustion conditions. Canadian Journal of Physiology and Pharmacology. 1972;50(8):761–7.
20. Graves BM, Johnson TJ, Nishida RT, Dias RP, Savareear B, Harynuk JJ, et al. Comprehensive characterization of mainstream marijuana and tobacco smoke. Scientific Reports. 2020;10(1):7160.
21. Lee M, Novotny M, Bartle K. Gas chromatography/mass spectrometric and nuclear magnetic resonance spectrometric studies of carcinogenic polynuclear aromatic hydrocarbons in tobacco and marijuana smoke condensates. Analytical Chemistry. 1976;48(2):405–16.
22. Tjeerdema R. The pyrolysis of cannabinoids. Reviews of Environmental Contamination and Toxicology 991987. p. 61–81.
23. Sparacino CM, Hyldburg PA, Hughes TJ. Chemical and biological analysis of marijuana smoke condensate. Research Findings on Smoking of Abused Substances. 991990. p. 121–40.
24. Küppers F, Bercht C, Salemink C, Lousberg R. *Cannabis*. XIV. Pyrolysis of cannabidiol—analysis of the volatile constituents. Journal of Chromatography A. 1975;108(2):375–9.
25. Küppers F, Bercht C, Salemink C, Lousberg RC, Terlouw J, Heerma W. *Cannabis*—XV: Pyrolysis of cannabidiol. Structure elucidation of four pyrolytic products. Tetrahedron. 1975;31(13-14):1513–6.
26. Küppers F, Lousberg RC, Bercht C, Salemink C, Terlouw J, Heerma W, et al. *Cannabis*—VIII: pyrolysis of Cannabidiol. Structure elucidation of the main pyrolytic product. Tetrahedron. 1973;29(18):2797–802.
27. Luteyn J, Spronck H, Salemink C. *Cannabis* XVIII: Isolation and synthesis of olivetol derivatives formed in the pyrolysis of cannabidiol. Recueil des Travaux Chimiques des Pays-Bas. 1978;97(7-8):187–90.
28. Mikeš F, Waser PG. Marihuana components: Effects of smoking on Δ^9-tetrahydrocannabinol and cannabidiol. Science. 1971;172(3988):1158–9.
29. Salemink CA. Pyrolysis of Cannabinoids. In: Nahas GG, editor. Marihuana: Chemistry, Biochemistry, and Cellular Effects: Springer-Verlag New York Inc.; 1976. p. 31–8.
30. Spronck H, Lousberg RC. Pyrolysis of cannabidiol. Structure elucidation of a major pyrolytic conversion product. Experientia. 1977;33(6):705–6.
31. Spronck H, Luteijn JM, Salemink CA, Nugteren DH. Inhibition of prostaglandin biosynthesis by derivatives of olivetol formed under pyrolysis of cannabidiol. Biochemical Pharmacology. 1978;27(4):607–8.
32. Spronck H, Salemink C. *Cannabis* XVII: Pyrolysis of cannabidiol. Structure elucidation of two pyrolytic conversion products. Recueil des Travaux Chimiques des Pays-Bas. 1978;97(7-8):185–6.
33. Bell S, Nida C. Pyrolysis of drugs of abuse: A comprehensive review. Drug Testing and Analysis. 2015;7(6):445–56.
34. Baker R. Temperature variation within a cigarette combustion coal during the smoking cycle. High Temp Sci. 1975;7:236–47.

35. Czégény Z, Nagy G, Babinszki B, Bajtel Á, Sebestyén Z, Kiss T, et al. CBD, a precursor of THC in e-cigarettes. Scientific Reports. 2021;11(1):8951.
36. Górecki T, Harynuk J, Panić O. The evolution of comprehensive two-dimensional gas chromatography (GC× GC). Journal of Separation Science. 2004;27(5-6):359–79.
37. Mondello L, Tranchida PQ, Dugo P, Dugo G. Comprehensive two-dimensional gas chromatography-mass spectrometry: A review. Mass Spectrometry Reviews. 2008;27(2):101–24.
38. Ubukata M, Kubo A, Nagatomo K, Hizume T, Ishioka H, Dane AJ, et al. Integrated qualitative analysis of polymer sample by pyrolysis–gas chromatography combined with high-resolution mass spectrometry: Using accurate mass measurement results from both electron ionization and soft ionization. Rapid Communications in Mass Spectrometry. 2020;34(15):e8820.
39. Cody RB, Fukudome T, Ubukata M. Integrated data analysis making use of the total information from gas chromatography and high-resolution time-of-flight mass spectrometry to identify qualitative differences between two whisky samples. Rapid Communications in Mass Spectrometry. 2022;36(3):e9225.
40. Kubo A, Kubota A, Ishioka H, Hizume T, Ubukata M, Nagatomo K. Construction of a mass spectrum library containing predicted electron ionization mass spectra prepared using a machine learning model and the development of an efficient search method. Mass Spectrometry. 2023;12(1):A0120–A.
41. Kubota A, Satoh T, Ubukata M, Kubo A, Nakayama C. Characterization of ultraviolet-degraded polyethylene terephthalate film using a complementary approach: Reactive pyrolysis–gas chromatography–mass spectrometry and matrix-assisted laser desorption/ionization mass spectrometry. Mass Spectrometry. 2025;14(1):A0168–A.
42. Zellner BdA, Bicchi C, Dugo P, Rubiolo P, Dugo G, Mondello L. Linear retention indices in gas chromatographic analysis: A review. Flavour and Fragrance Journal. 2008;23(5):297–314.
43. Developmental toxicity of two trimethylbenzene isomers, mesitylene and pseudocumene, in rats following inhalation exposure. Food and Chemical Toxicology. 2005;43(7):1055–63.
44. Agency EP. Provisional Peer-Reviewed Toxicity Values for Methylcyclopentane. 2009.
45. Baldissera MD, Souza CF, Dolci GS, Grando TH, Sagrillo MR, Vaucher RA, et al. Monoterpene alpha-terpinene induced hepatic oxidative, cytotoxic and genotoxic damage is associated to caspase activation in rats. Journal of Applied Biomedicine. 2017;15(3):187–95.
46. Bingham E, Cohrssen B, Powell C. Patty's toxicology volumes 1–9. 5th John Wiley & Sons2001. 703 p.
47. Cancer IAfRo. IARC monographs on the evaluation of carcinogenic risks to humans. Some industrial chemicals. Volume 60. World Health Organization; 1994.
48. Cancer IAfRo. Dry cleaning, some chlorinated solvents and other industrial chemicals. IARC monographs on the evaluation of carcinogenic risks to humans. Volume 63. World Health Organization; 1996. Report No.: 0957-5243.
49. Dean B. Genetic toxicology of benzene, toluene, xylenes and phenols. Mutation Research/Reviews in Genetic Toxicology. 1978;47(2):75–97.
50. Gagnaire F, Langlais C. Relative ototoxicity of 21 aromatic solvents. Archives of Toxicology. 2005;79(6):346–54.

51. Henderson L, Brusick D, Ratpan F, Veenstra G. A review of the genotoxicity of ethylbenzene. Mutation Research/Reviews in Mutation Research. 2007;635(2-3):81–9.
52. Hoang NMH, Jo W, Kim M-S. Protective effect of Prolactin releasing peptide against 1, 2-diacetylbenzene-induced neuroinflammation. Neuropeptides. 2023;100:102349.
53. Hoang NMH, Kim S, Nguyen HD, Kim M, Kim J, Kim B-C, et al. Age-dependent sensitivity to the neurotoxic environmental metabolite, 1, 2-diacetylbenzene. Biomolecules & Therapeutics. 2021;29(4):399.
54. Janik-Spiechowicz E, Wyszyńska K. Genotoxicity of Farbasol and its component: 4-ethyltoluene. Mutation Research/Genetic Toxicology and Environmental Mutagenesis. 1998;417(2-3):95–100.
55. Janik-Spiechowicz E, Wyszynska K, Dziubałtowska E. Genotoxicity evaluation of trimethylbenzenes. Mutation Research/Genetic Toxicology and Environmental Mutagenesis. 1997;412(3):299–305.
56. Korsak Z, Stetkiewicz J, Majcherek W, Stetkiewicz I, Jajte J, Rydzynski K. Sub-chronic inhalation toxicity of 1, 2, 4-trimethylbenzene (pseudocumene) in rats. International Journal of Occupational Medicine and Environmental Health. 2000;13(2):155–64.
57. Korsak Z, Swiercz R, Rydzynski K. Toxic effects of acute inhalation exposure to 1, 2, 4-trimethylbenzene (pseudocumene) in experimental animals. International Journal of Occupational Medicine and Environmental Health. 1995;8(4):331–7.
58. Liewen MB, Marth EH. Evaluation of 1, 3-pentadiene for mutagenicity by the *Salmonella*/mammalian microsome assay. Mutation Research/Genetic Toxicology. 1985;157(1):49–52.
59. Maltoni C, Ciliberti A, Pinto C, Soffritti M, Belpoggi F, Menarini L. Results of long-term experimental carcinogenicity studies of the effects of gasoline, correlated fuels, and major gasoline aromatics on rats. Annals of the New York Academy of Sciences. 1997;837(1):15–52.
60. Norppa H, Vainio H. Induction of sister-chromatid exchanges by styrene analogues in cultured human lymphocytes. Mutation Research/Genetic Toxicology. 1983;116(3-4):379–87.
61. Norseth T, Waage J, Dale I. Acute effects and exposure to organic compounds in road maintenance workers exposed to asphalt. American Journal of Industrial Medicine. 1991;20(6):737–44.
62. Program NT. Toxicology and carcinogenesis studies of alpha-methylstyrene (Cas No. 98-83-9) in F344/N rats and B6C3F1 mice (inhalation studies). National Institute of Health; 2007. Report No.: 0888-8051 Contract No.: 543.
63. Saillenfait A, Gallissot F, Sabate J, Morel G. Developmental toxicity of two trimethylbenzene isomers, mesitylene and pseudocumene, in rats following inhalation exposure. Food and Chemical Toxicology. 2005;43(7):1055–63.
64. Świercz R, Rydzyński K, Jajte J, Stetkiewicz J, Majcherek W. Studies on dermal, ocular and respiratory effects of 4-ethyltoluene in experimental animals. International Journal of Occupational Medicine and Environmental Health. 2000;13(4):307–15.
65. Registry AfTSaD. Toxicological Profile for *n*-Hexane. U.S. Department of Health and Human Services; 2025.
66. Wiaderna D, Gralewicz S, Tomas T. Behavioural changes following a four-week inhalation exposure to hemimellitene (1, 2, 3-trimethylbenzene) in rats. International Journal of Occupational Medicine and Environmental Health. 1998;11(4):319–34.

67. Wojtunik-Kulesza KA. Toxicity of selected monoterpenes and essential oils rich in these compounds. Molecules. 2022;27(5):1716.
68. Yardley-Jones A, Anderson D, Parke D. The toxicity of benzene and its metabolism and molecular pathology in human risk assessment. Occupational and Environmental Medicine. 1991;48(7):437–44.
69. Zhang L-J, Feng W-T, Liu J-J. Progress on the mechanism of *n*-hexane induced toxic effects in vitro and in vivo. Chinese Journal of Industrial Hygiene and Occupational Diseases. 2023;41(5):388–96.
70. Galvin JB, Bond G. 2-Methylpentane (isohexane). Journal of Toxicology and Environmental Health Part A. 1999;58(1-2):81–92.
71. Gibbs BF, Mulligan CN. Styrene toxicity: An ecotoxicological assessment. Ecotoxicology and Environmental Safety. 1997;38(3):181–94.
72. Korsak Z, Rydzynski K. Neurotoxic effects of accute and subchronic inhalation exposure to trimethylbenzene isomers (pseudocumene, mesitylene, hemimellitene) in rats. International Journal of Occupational Medicine and Environmental Health. 1996;9:341–50.
73. Roberts GK, Willson CJ, Olivera DS, Malarkey DE, Morgan DL. Comparative inhalation toxicity of ethyltoluene isomers in rats and mice. Inhalation Toxicology. 2017;29(12-14):577–85.
74. Surendran S, Qassadi F, Surendran G, Lilley D, Heinrich M. Myrcene—what are the potential health benefits of this flavouring and aroma agent? Frontiers in Nutrition. 2021;8:1–14.
75. Hard GC, Whysner J. Risk assessment of *D*-limonene: An example of male rat-specific renal tumorigens. Critical Reviews in Toxicology. 1994;24(3):231–54.
76. AlSaffar RM, Rashid S, Ahmad SB, Rehman MU, Hussain I, Parvaiz Ahmad S, et al. *D*-limonene (5 (one-methyl-four-[1-methylethenyl]) cyclohexane) diminishes CCl4-induced cardiac toxicity by alleviating oxidative stress, inflammatory and cardiac markers. Redox Report. 2022;27(1):92–9.
77. Sun J. *D*-Limonene: Safety and clinical applications. Alternative Medicine Review. 2007;12(3):259.
78. Andrade VA, Almeida AC, Souza DS, Colen KG, Macêdo AA, Martins ER, et al. Antimicrobial activity and acute and chronic toxicity of the essential oil of *Lippia origanoides*. Pesquisa Veterinária Brasileira. 2014;34:1153–61.
79. Marques FM, Figueira MM, Schmitt EFP, Kondratyuk TP, Endringer DC, Scherer R, et al. In vitro anti-inflammatory activity of terpenes via suppression of superoxide and nitric oxide generation and the NF-κB signalling pathway. Inflammopharmacology. 2019;27(2):281–9.
80. Marchese A, Arciola CR, Barbieri R, Silva AS, Nabavi SF, Tsetegho Sokeng AJ, et al. Update on monoterpenes as antimicrobial agents: A particular focus on *p*-cymene. Materials. 2017;10(8):947.
81. Alali F, Al-Lafi T. GC-MS analysis and bioactivity testing of the volatile oil from the leaves of the toothbrush tree *Salvadora persica L.* Natural Product Research. 2003;17(3):189–94.
82. Aydin E, Türkez H, Taşdemir Ş. Anticancer and antioxidant properties of terpinolene in rat brain cells. Archives of Industrial Hygiene and Toxicology. 2013;64(3):415–24.

83. Baginska S, Golonko A, Swislocka R, Lewandowski W. Monoterpenes as medicinal agents: Exploring the pharmaceutical potential of *p*-cymene, *p*-cymenene, and *γ*-terpinene. Acta Poloniae Pharmaceutica. 2023;80(6):879–92.
84. Di Napoli M, Castagliuolo G, Badalamenti N, Vaglica A, Ilardi V, Varcamonti M, et al. Chemical composition, antimicrobial and antioxidant activities of the essential oil of Italian *Prangos trifida* (Mill.) Herrnst. & Heyn. Natural Product Research. 2023;37(22):3772–86.
85. Flach A, Gregel B, Simionatto E, da Silva UF, Zanatta N, Morel AF, et al. Chemical analysis and antifungal activity of the essential oil of *Calea clematidea*. Planta Medical 2002;68(09):836–8.
86. Hashemi M, Ehsani A, Jazani NH, Aliakbarlu J, Mahmoudi R. Chemical composition and in vitro antibacterial activity of essential oil and methanol extract of *Echinophora platyloba* DC against some of food-borne pathogenic bacteria. Veterinary Research Forum. 2013;4(2):123.
87. Nasir Shah S, Khan I, Tul Muntaha S, Hayat A, Ur Rehman M, Ali Shah T, et al. Bioactive, antioxidant and antimicrobial properties of chemically fingerprinted essential oil s extracted from *Eucalyptus globulus*: In-vitro and in-silico investigations. Frontiers in Chemistry. 2023;11:1287317.
88. Prasher P, Sharma M. Medicinal chemistry of indane and its analogues: A mini review. Chemistry Select. 2021;6(11):2658–77.
89. Shafaghat A. Antimicrobial activity and volatile constituents of the essential oil of *Pulsatilla albana* from Iran. Natural Product Communications. 2010;5(8):1299–300.
90. Zubkov FI, Kouznetsov VV. Traveling across life sciences with acetophenone—a simple ketone that has special multipurpose missions. Molecules. 2023;28(1):370.
91. Api A, Belsito D, Botelho D, Bruze M, Burton Jr G, Buschmann J, et al. RIFM fragrance ingredient safety assessment, acetophenone, CAS Registry Number 98-86-2. 2018. Report No.: 1873-6351.
