# Supplementary material for: Detection and identification of small-molecule cracking products formed on pyrolysis of cannabidiol part 1: C3 – C10 compounds
Source: J Cannabis Res. 2026 May 1;8:90. doi: 10.1186/s42238-026-00442-8 (PMC13445818; doi:10.1186/s42238-026-00442-8)
Supplement: Supplementary file 3 [file 42238_2026_442_MOESM1_ESM.docx]

| **Table S1.** Standards used for compound identity confirmation and the corresponding suppliers. | |
| --- | --- |
| **Compound Name** | **Vendor** |
| Cyclopropane | Sigma-Aldrich |
| *(Z)-*2-Butene | Sigma-Aldrich |
| 1,3-Pentadiene | TCI America |
| 2-Methylpentane | Beantown Chemical Corporation |
| *n*-Hexane | Sigma-Aldrich |
| *(Z)-*3-Methyl-2-pentene | Aladdin Scientific |
| 2,3-Dimethyl-1,3-butadiene | TCI America |
| Methylcyclopentane | TCI America |
| 1-Methylcyclopentene | Thermo Scientific |
| Benzene | Sigma-Aldrich |
| 1,3-Cyclohexadiene | Thermo Scientific |
| *(Z)-*3,4-Dimethyl-2-pentene | TCI America |
| *(Z)-*3-Methyl-3-hexene | Sigma-Aldrich |
| Toluene | Sigma-Aldrich |
| 1-Methylcyclohexa-1,3-diene | Advanced ChemBlock Inc |
| 1,3-Cycloheptadiene | Sigma-Aldrich |
| Ethylbenzene | Thermo Scientific |
| *p*-Xylene | Sigma-Aldrich |
| *m*-Xylene | Beantown Chemical Corporation |
| Styrene | Alfa Chemicals |
| *o*-Xylene | Thermo Scientific |
| 1-Ethyl-4-methylbenzene | TCI America |
| 1-Ethyl-3-methylbenzene | AmBeed |
| 1,3,5-Trimethylbenzene | Beantown Chemical Corporation |
| 1-Ethyl-2-methylbenzene | Sigma-Aldrich |
| Phenol | Thermo Scientific |
| α-Methylstyrene | TCI America |
| β-Myrcene | Sigma-Aldrich |
| 1,2,4-Trimethylbenzene | Sigma-Aldrich |
| 1-Ethenyl-2-methylbenzene | AmBeed |
| 1-Ethenyl-3-methylbenzene | Sigma-Aldrich |
| Benzofuran | TCI America |
| 1-Ethenyl-4-methylbenzene | Sigma-Aldrich |
| 1,2,3-Trimethylbenzene | TCI America |
| 1-Propenylbenzene | Thermo Scientific |
| *o*-Cymene | Sigma-Aldrich |
| *m*-Cymene | TCI America |
| *D*-Limonene | Sigma-Aldrich |
| **Table S1 (continued).** Standards used for compound identity confirmation and the corresponding suppliers. | |
| **Compound Name** | **Vendor** |
| Indane | AmBeed |
| *p*-Cymene | Thermo Scientific |
| 1-Propynylbenzene | Thermo Scientific |
| 1-Methyl-2-propylbenzene | TCI America |
| 1-Ethynyl-4-methylbenzene | Apollo Scientific |
| 1,2-Diethylbenzene | Thermo Scientific |
| Acetophenone | Beantown Chemical Corporation |
| 1-Ethyl-2,4-dimethylbenzene | TCI America |
| 3-Methyl-phenol | TCI America |
| 1-Methyl-3-(1-methylethenyl)-benzene | AmBeed |
| 1-Methyl-4-(1-methylethylidene)-cyclohexene | Sigma-Aldrich |
| 1-Methyl-4-(1-methylethenyl)-benzene | TCI America |
| 2-Ethynl-1,4-dimethylbenzene | AmBeed |
| 2-Methyl-benzofuran | AmBeed |
